# Molecular Hydrogen Attenuates Neuropathic Pain in Mice

**DOI:** 10.1371/journal.pone.0100352

**Published:** 2014-06-18

**Authors:** Masanori Kawaguchi, Yasushi Satoh, Yukiko Otsubo, Tomiei Kazama

**Affiliations:** Department of Anesthesiology, National Defense Medical College, Tokorozawa, Saitama, Japan; University of Texas Medical Branch, United States of America

## Abstract

Neuropathic pain remains intractable and the development of new therapeutic strategies are urgently required. Accumulating evidence indicates that overproduction of oxidative stress is a key event in the pathogenesis of neuropathic pain. However, repeated intra-peritoneal or intrathecal injections of antioxidants are unsuitable for continuous use in therapy. Here we show a novel therapeutic method against neuropathic pain: drinking water containing molecular hydrogen (H_2_) as antioxidant. The effect of hydrogen on neuropathic pain was investigated using a partial sciatic nerve ligation model in mice. As indicators of neuropathic pain, temporal aspects of mechanical allodynia and thermal hyperalgesia were analysed for 3 weeks after ligation. Mechanical allodynia and thermal hyperalgesia were measured using the von Frey test and the plantar test, respectively. When mice were allowed to drink water containing hydrogen at a saturated level *ad libitum* after ligation, both allodynia and hyperalgesia were alleviated. These symptoms were also alleviated when hydrogen was administered only for the induction phase (from day 0 to 4 after ligation). When hydrogen was administered only for the maintenance phase (from day 4 to 21 after ligation), hyperalgesia but not allodynia was alleviated. Immunohistochemical staining for the oxidative stress marker, 4-hydroxy-2-nonenal and 8-hydroxydeoxyguanosine, showed that hydrogen administration suppressed oxidative stress induced by ligation in the spinal cord and the dorsal root ganglion. In conclusion, oral administration of hydrogen water may be useful for alleviating neuropathic pain in a clinical setting.

## Introduction

Neuropathic pain is one of abnormal chronic pain that is produced after trauma or disease of the peripheral or the central nervous system. The common symptoms of neuropathic pain are mechanical allodynia, a painful response to innocuous tactile stimuli, and hyperalgesia, a decrease in nociceptive thresholds to the stimuli. Neuropathic pain remains intractable and many patients suffering from this disease exact a tremendous cost to the economy. Anti-inflammatories including non-steroidal anti-inflammatory drug and some narcotic analgesics are not effective for managing neuropathic pain and the need for higher dosages of these drugs leads to unsatisfactory side effects. Therefore, the development of new therapeutic strategies is urgently required.

Reactive oxygen species (ROS) are reported to be involved in the pathomechanism of neuropathic pain [1]–[4]. Although the mechanism by which excessive ROS produce pain is largely unclear, antioxidants have received attention as novel analgesics against neuropathic pain [3]. It was reported that systemic administration of antioxidants produced an analgesic effect in animal models of neuropathic pain. For instance, phenyl-*N-tert*-butylnitrone (PBN), 5,5-dimethylpyrroline-N-oxide, vitamin E, and 4-hydroxy-2,2,6,6-tetramethylpiperidine-1-oxyl (TEMPOL) could ameliorate the behavioral signs of neuropathic pain [5]–[8]. In these reports, analgesia was extended by repeated injections of drugs at short intervals. Thus, if these agents are to be useful in therapy, they will have to be given continuously or at relatively short interval, which is unsuitable for clinical medication.

Molecular hydrogen has recently received attention as a novel antioxidant with high efficacy and no currently known side effect [9]. Because of its small size and electrically neutral properties, molecular hydrogen can reach target organs easily. Drinking water with dissolved hydrogen has been reported to be effective in preventing oxidative stress in some animal models of other diseases [10]–[12]. In addition, hydrogen does not accumulate in living cells nor produce noxious metabolites, which sometimes cause distress in other antioxidants.

ROS include hydroxyl radical, hydrogen peroxide, superoxide, nitric oxide, and nitroperoxide. Among them, hydroxyl radical is extremely toxic and has no currently known beneficial role. Furthermore, no defense system against hydroxyl radical has been found in mammalian cells. On the other hand, hydrogen peroxide, superoxide, and nitric oxide have important roles in various biological processes at low concentrations [13]–[15]. Thus, there is a concern about continuous use of antioxidant that might cause serious side effect. Hydrogen is a more attractive antioxidant, in that unlike other antioxidants it can selectively neutralize the hydroxyl radical, while having minimal effect on beneficial ROS [9].

In the present study, we show that molecular hydrogen can ameliorate mechanical allodynia and thermal hyperalgesia. Our results indicate that hydrogen administration by the oral pathway may have potential for the management of neuropathic pain.

## Materials and Methods

### Ethics Statement

The care and use of all mice in this study was carried out in accordance with institutional ethical guidelines for animal experiments of the National Defense Medical College (Tokorozawa, Saitama, Japan), and were approved by the Committee for Animal Research at the National Defense Medical College. Guidelines of the Committee for Research and Ethical Issues of the International Association for the Study of Pain [16] was strictly adhered to in this study.

### Animals

We used C57BL/6 mice at 8-weeks old when subjected to nerve ligation. These mice were maintained on a 12-h light–dark cycle (lights on from 07∶00 to 19∶00) with room temperature at 21±1°C. They had access to water and food *ad libitum*.

### Hydrogen Water

Hydrogen water was produced by Blue Mercury Inc. (Tokyo, Japan), which contained more than 0.6 mM hydrogen. Hydrogen water was put in an aluminium bottle (60 ml) to maintain the concentration of hydrogen. Mice were allowed to drink hydrogen water *ad libitum* for indicated time. Hydrogen water was exchanged every day to keep the hydrogen concentration constant. Control groups were supplied with distilled water. There was no significant difference in the amounts of water consumed between hydrogen group and control group (3.87±0.13 vs. 3.91±0.15 ml, mean ± SEM, n = 12 mice for each). Amount of water consumed was monitored by recording the volume of water in the bottle.

### Mouse Study Design

Siblings from the same litter were randomly allocated into one of the following groups so that each group was balanced on littermate. Ligation group: n = 12, Ligation+hydrogen (day 0–21) group: n = 12, Ligation+hydrogen (day 0–4) group: n = 12, Ligation+hydrogen (day 4–21) group: n = 12, Sham group: n = 12, Sham+hydrogen (day 0–21) group: n = 12.

### Nerve Injury Pain Model

Throughout the experiment, we used a partial sciatic nerve ligation (PSNL) model as a neuropathic pain model (Seltzer model) as described previously [17]. Briefly, the sciatic nerve located in the right hind paw was exposed via a small incision and approximately 1/3–1/2 the diameter of the nerve was tightly ligated with an 8–0 silk suture. PSNL caused a fast and pronounced development of mechanical allodynia and thermal hyperalgesia in mice. In the sham-operated groups, sciatic nerves were exposed without ligation. The pain behaviors were assessed by the von Frey test and the plantar test for mechanical allodynia and thermal hyperalgesia, respectively. These behaviors were tested before (Pre) and after PSNL operation (day 1, 4, 7, 14, and 21). Measurement was carried out by an investigator blinded to the hydrogen treatment.

### Measurement of Mechanical Allodynia

To quantify mechanical allodynia, paw withdrawal responses to mechanical stimulation were assessed with von Frey filaments as previously described [17]. Briefly, mice were habituated for at least 1 h prior to testing in a clear acrylic cylinder (20 cm in height and 10 cm in diameter) on an elevated mesh floor. Calibrated von Frey filaments (North Coast Medical, Gilroy, CA) were then applied through the mesh to the plantar surface on hind paw. Filaments were applied for 1 s and this was repeated 5 times at a frequency of 0.5 Hz. Filaments were pressed until the filament bent. Paw movements associated with locomotion or weight shifting were not counted as responses. The lightest filament of 0.008 g bending force was initially applied with progressively increasing force (0.02, 0.04, 0.07, 0.16, 0.4, 0.6, 1.0, 1.4, and 2.0 g). Measurement was finished when paw withdrawals were evoked at least three withdrawals out of five applications, and the calibrated bending force of the filament was defined as the paw withdrawal threshold.

### Measurement of Thermal (Heat) Hyperalgesia

Response to thermal stimulation were measured as previously described [18] with some modifications using a plantar test apparatus (ANESTHESIA RESEARCH Lab, University of California, San Diego, CA). The intensity of the thermal stimulus was adjusted to achieve a baseline paw-withdrawal latency of approximately 7–9 sec on average in naive mice. Paw withdrawal latency was determined as the average of three scores per paw.

### Immunohistochemical Studies

Immunohistochemical study on the dorsal root ganglion (DRG) and the spinal cord of at the lumbar level was performed as previously described [19]. Briefly, paraffin sections (5 µm thick) were deparaffinized and immersed in unmasking solution (Vector H3300; Vector Laboratories, Burlingame, CA) for antigenic retrieval and heated in an autoclave (121°C) for 5 min. The sections were then incubated in a nonspecific blocking reagent (Dako, Glostrup, Denmark) for 30 min to reduce background staining. Sections were then incubated with primary antibodies overnight in a humidified chamber at 4°C. Primary antibodies used in this study were anti-4-hydroxy-2-nonenal (4-HNE) (mouse monoclonal, Japan Institute for the Control of Aging, Shizuoka, Japan), anti-8-hydroxydeoxyguanosine (8-OHdG) (mouse monoclonal, Japan Institute for the Control of Aging), anti-neuronal nuclear antigen (NeuN) (rabbit polyclonal, Millipore, Billerica, MA), anti-glial fibrillary acidic protein (GFAP) (rabbit polyclonal, Thermo Fisher Scientific, Waltham, MA), anti-NG2 (rabbit polyclonal, Abcam, Cambridge, UK), anti-Olig2 (rabbit polyclonal, Abcam), and anti-Iba1 (rabbit polyclonal, Wako, Osaka, Japan) antibodies.

For fluorescent staining, sections were incubated with Alexa-Fluor 488-conjugated goat anti-rabbit IgG (1∶200; Molecular Probes, Eugene, OR) for primary antibodies derived from mouse. For primary antibody derived from rabbit, Alexa-Fluor 546-conjugated goat anti-mouse IgG (1∶200; Molecular Probes) was used. Sections were examined using a Nikon fluorescence microscopy system (Nikon, Tokyo, Japan) with electron-multiplying (EM) CCD digital camera (ImagEM, Hamamatsu Photonics, Hamamatsu, Japan).

Samples from six slides (from six mice) per experimental conditions were examined. For quantification of the spinal cord sections, total number of positive cells for 4-HNE or 8OHdG was counted. For quantification of the DRG sections, the proportion of positive cells was determined by positive neuron profile divided by the total neuronal profiles on each section. Counting of immunostaining was performed by an investigator blinded to the treatment conditions.

### Statistics

Statistical analysis was performed using GraphPad Prism 5 (GraphPad Software Inc.). Comparisons of the means of each group were performed using a Student’s *t* test or two-way repeated measures analysis of variance (two-way RM ANOVA) with a Bonferroni post-hoc test. Details regarding specific statistical tests are included in the results. Values are presented as the mean ± SEM.

## Results

### Mechanical Allodynia and Hyperalgesia Induced by PSNL was Alleviated by Hydrogen Water

**Figure 1:**
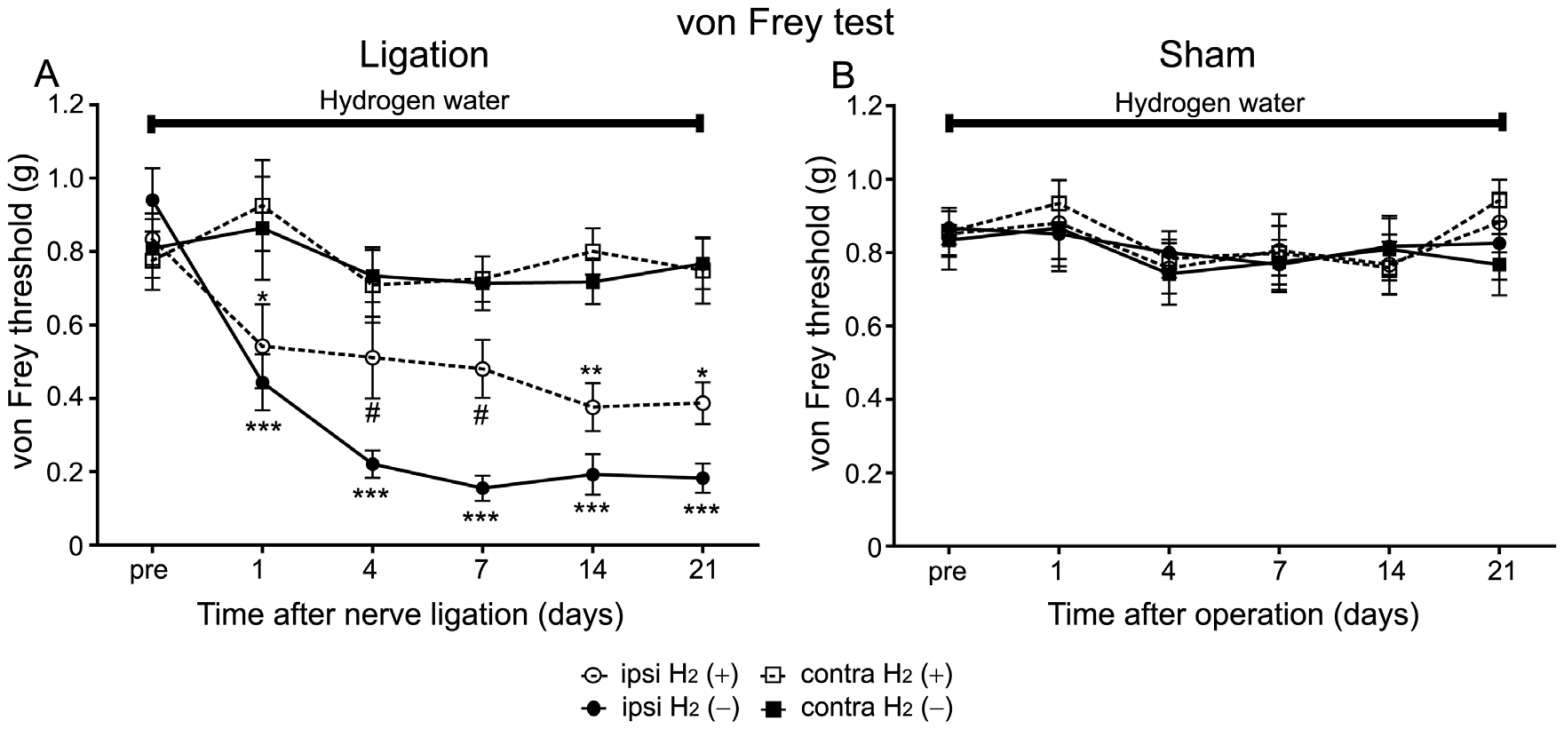
Hydrogen treatment suppressed mechanical allodynia in the PSNL model. Neuropathic mechanical allodynia was analysed by the von Frey test. Hydrogen was supplied for the whole experimental period right after PSNL or sham operation. The control group received distilled water from day 0 to 21. (A) The hydrogen-treated mice exhibited higher threshold on the ipsilateral side to ligation than controls (n = 12 mice for each group). (B) Sham operation did not caused mechanical allodynia regardless of hydrogen treatment (n = 12 mice for each group). We used a two-way RM ANOVA with a Bonferroni post-hoc test; *P<0.05, **P<0.01, ***P<0.001 vs. contralateral side of control mice, #P<0.05 vs. ipsilateral side of control mice.

To examine the effect of hydrogen water against neuropathic pain, we tested whether *ad libitum* drinking of hydrogen water modified mechanical allodynia and hyperalgesia in PSNL model mice. In control mice (without hydrogen), PSNL caused a significant and long-lasting decrease in their mechanical threshold measured with von Frey hairs (Figure 1A). A two-way RM ANOVA confirmed this, indicating a significant difference between the ipsilateral and the contralateral sides to ligation (*F* = 91.46, *P*<0.0001). On the other hand, in the sham operated group, there was no significant difference in mechanical thresholds between the ipsilateral and the contralateral sides to operation (Figure 1B; two-way RM ANOVA, ipsilateral vs. contralateral, *P*>0.05).

When mice were supplied with hydrogen water for the whole experimental period (from day 0 (right after PSNL operation) to 21), they exhibited a significant and long-lasting decrease in their mechanical threshold on the ipsilateral side to ligation compared with the contralateral side (Figure 1A; two-way RM ANOVA, ipsilateral vs. contralateral, *F* = 16.21, *P*<0.001), similar to mice without hydrogen treatment. However, the hydrogen-treated mice exhibited higher threshold on the ipsilateral side to ligation when compared with mice without hydrogen treatment (Figure 1A). A two-way RM ANOVA indicated a significant difference between hydrogen-treated mice and controls (ipsilateral H_2_ (+) vs. ipsilateral H_2_ (–), *F* = 9.43, *P*<0.0046). In the sham-operated group with hydrogen treatment, there was no significant difference in mechanical thresholds between the ipsilateral and the contralateral sides to operation (Figure 1B; two-way RM ANOVA, ipsilateral vs. contralateral, *P*>0.05). These results indicate that hydrogen treatment significantly alleviated mechanical allodynia caused by PSNL.

**Figure 2:**
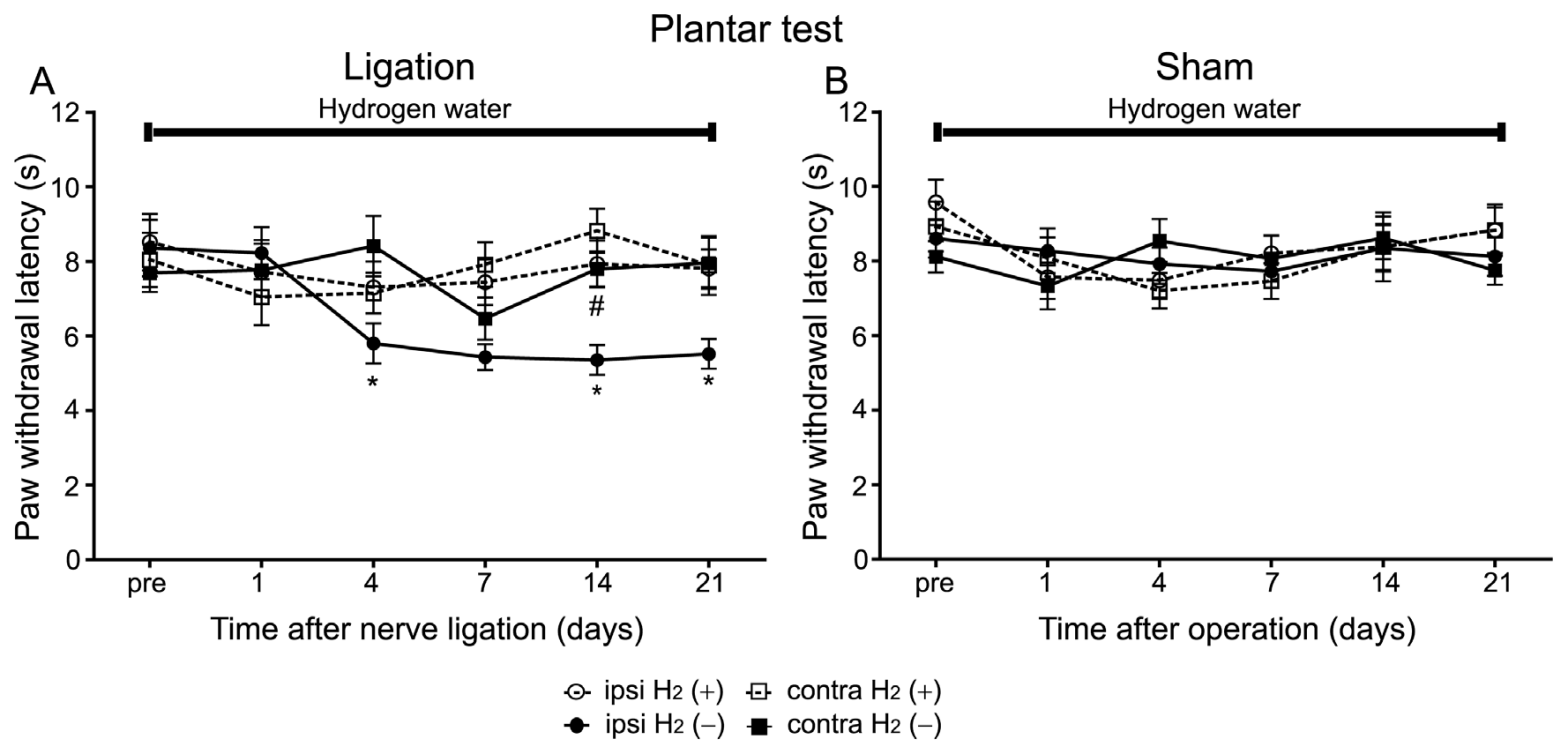
Hydrogen treatment suppressed thermal hyperalgesia in the PSNL model. Thermal hyperalgesia was analysed by the plantar test. The same sets of mice used for the von Frey test were used in the plantar test. (A) The reduction of paw withdrawal latency was attenuated in mice with hydrogen compared with those without hydrogen (n = 12 mice for each group). (B) Sham operation did not cause hyperalgesia regardless of hydrogen treatment (n = 12 mice for each group). We used a two-way RM ANOVA with a Bonferroni post-hoc test; *P<0.05 vs. contralateral side of control mice,^ #^ *P*<0.05 vs. ipsilateral side of control mice.

In the plantar test, control mice exhibited significant reduction of paw withdrawal latency only on the ipsilateral side to the ligation compared with the contralateral side (Figure 2A). A two-way RM ANOVA conformed this, indicating a significant difference between the ipsilateral and the contralateral sides to ligation (ipsilateral vs. contralateral, *F* = 13.36, *P* = 0.0014). On the other hand, in the sham operated group, there was no significant difference in paw withdrawal latency between the ipsilateral and the contralateral sides to operation (Figure 2B; two-way RM ANOVA, ipsilateral vs. contralateral, *P*>0.05).

When mice were supplied with hydrogen water for the whole experimental period, they did not exhibit significant reduction in paw withdrawal latency on the ipsilateral side to ligation compared with the contralateral side (Figure 2A; two-way RM ANOVA, ipsilateral vs. contralateral, *P*>0.05). Furthermore, paw withdrawal latency on the ipsilateral side of mice with hydrogen treatment was significantly longer than that of mice without hydrogen treatment (Figure 2A). A two-way RM ANOVA indicated a significant difference between them (ipsilateral H_2_ (+) vs. ipsilateral H_2_ (–), *F* = 11.42, *P* = 0.0027). In the sham-operated group with hydrogen treatment, there was no significant difference in paw withdrawal latency between the ipsilateral and the contralateral sides to operation (Figure 2B; two-way RM ANOVA, ipsilateral H_2_ (+) vs. contralateral H_2_ (+), *P*>0.05). These results indicate that both mechanical allodynia and thermal hyperalgesia induced by PSNL can be alleviated by drinking hydrogen water.

### Hydrogen Treatment was Effective against the Development Phase but not against the Maintenance Phase of Mechanical Allodynia

**Figure 3:**
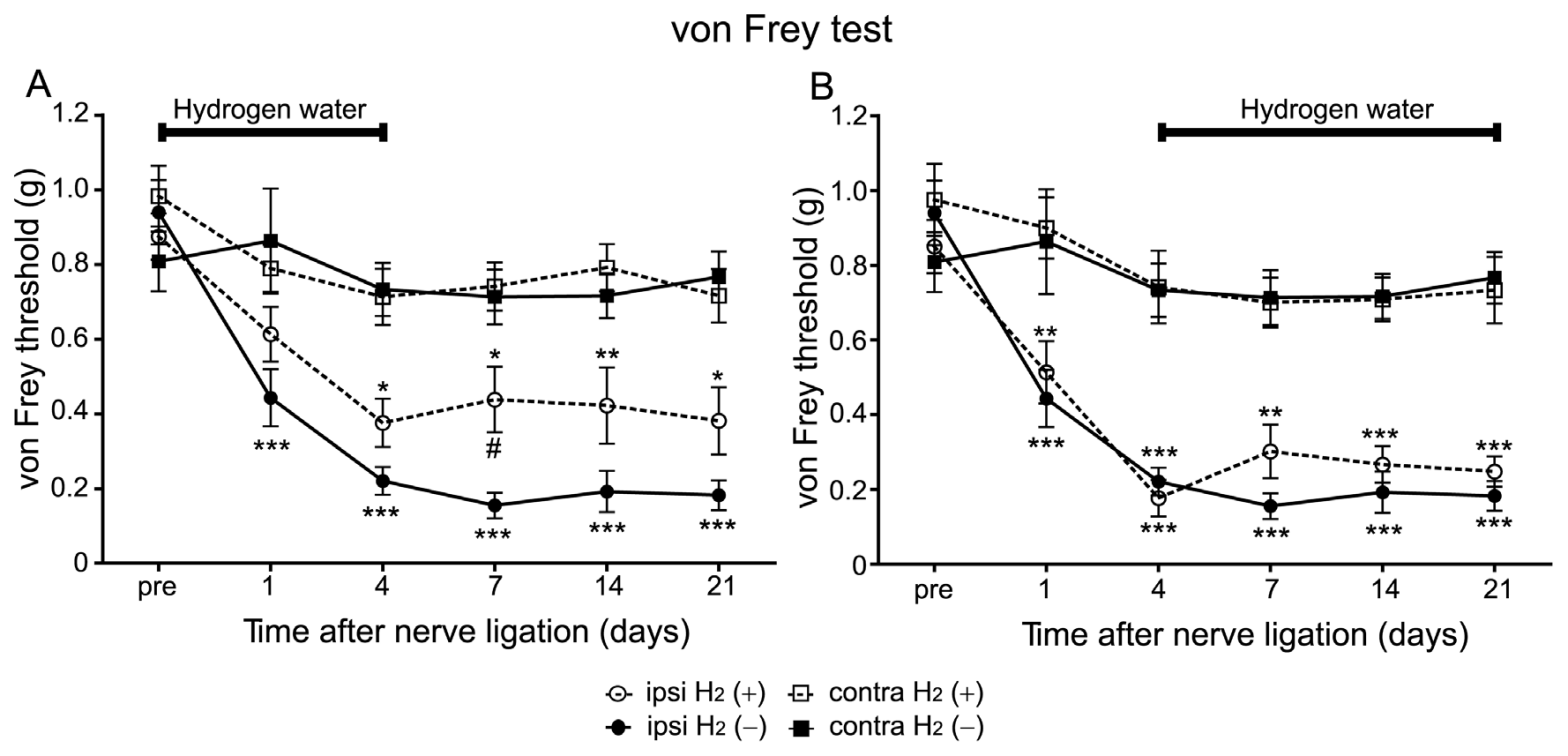
Hydrogen treatment was effective against the induction phase but not against the maintenance phase of mechanical allodynia. (A) Hydrogen water was supplied from day 0 to 4, and after then changed to distilled water. Paw withdrawal threshold was significantly lower in the H_2_ (+) group when compared with the H_2_ (–) group (n = 12 mice for each group). Note that the lower threshold in the H_2_ (+) group lasted beyond the termination of hydrogen treatment. (B) Hydrogen water was supplied from day 4 to 21. There was no significant difference in paw withdrawal threshold between the H_2_ (+) and the H_2_ (–) groups (n = 12 mice for each group). These experiments were performed concurrently with those in Figure 1. We used a two-way RM ANOVA with a Bonferroni post-hoc test; *P<0.05, **P<0.01, ***P<0.001 vs. contralateral side of same mice. ^#^ *P*<0.05 vs. ipsilateral side of H_2_ (–) mice.

In the Seltzer model, the time course of the pain responses consists of two-phases. The pain responses in the von Frey and the plantar test were gradually increased till 4 days after PSNL and then followed by sustained pain behaviors as observed in figures 1 and 2. To examine the effect of hydrogen on the induction phase, hydrogen was administered from day 0 to 4. The von Frey test revealed that mice with hydrogen treatment exhibited a higher threshold on the ipsilateral side when compared with that of mice without hydrogen (Figure 3A). A two-way RM ANOVA indicated a significant difference between hydrogen-treated mice and controls (ipsilateral H_2_ (+) vs. ipsilateral H_2_ (–), *F* = 9.06, *P* = 0.0064). These results indicate that hydrogen treatment was effective on the development of mechanical allodynia. It is worth noting that an analgesic effect lasted for the whole experiment period even after the termination of hydrogen treatment (Figure 3A).

When hydrogen was administered from day 4 to 21 (maintenance phase), the von Frey test indicated that there was no significant difference in mechanical thresholds on the ipsilateral side to ligation between mice with hydrogen treatment and controls (Figure 3B; two-way RM ANOVA, ipsilateral H_2_ (+) vs. ipsilateral H_2_ (–), *P*>0.05). These results indicate that hydrogen treatment is effective against the induction phase, but not against the maintenance phase in mechanical allodynia.

### Hydrogen Treatment was Effective against both Induction and Maintenance Phases in Thermal Hyperalgesia

**Figure 4:**
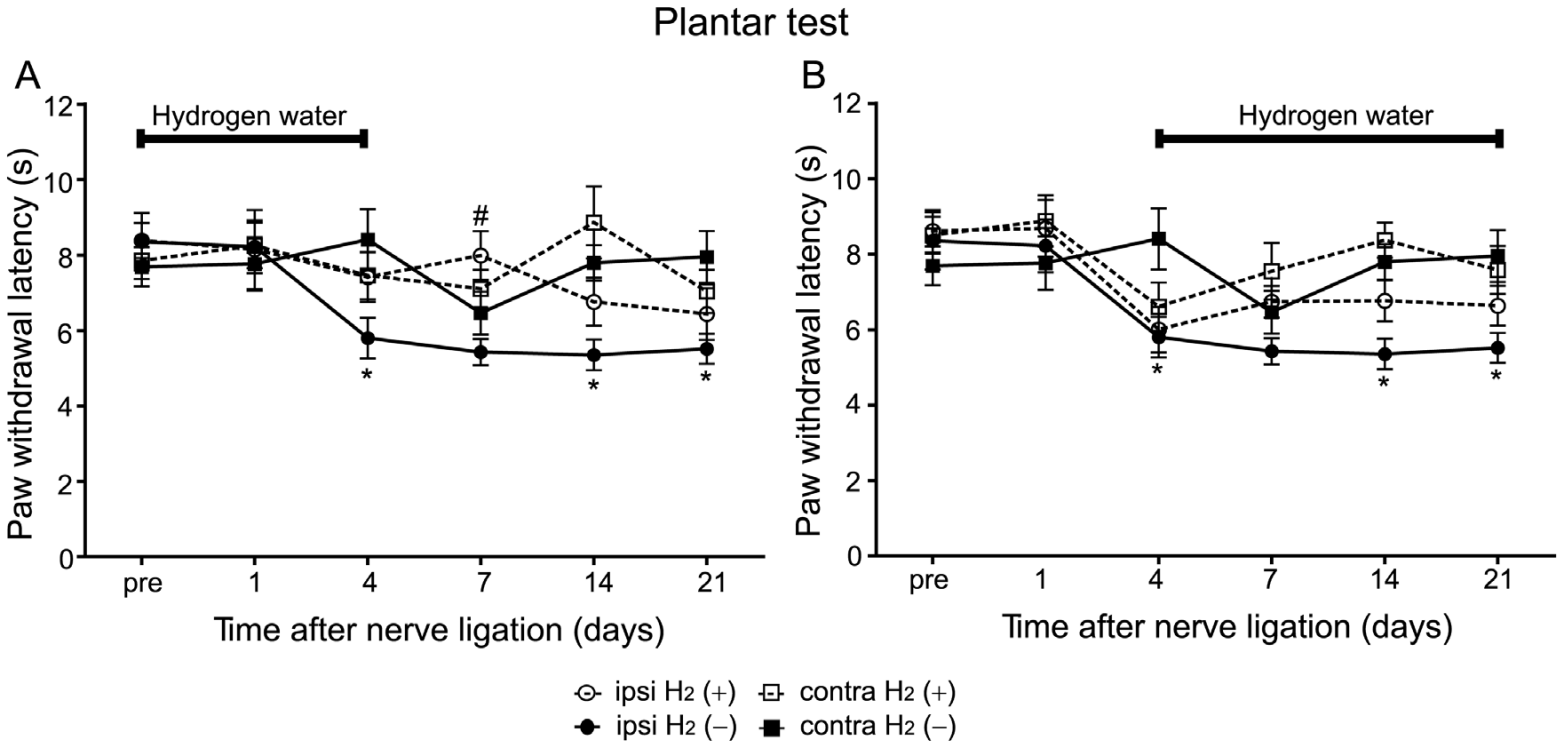
Hydrogen treatment was effective against both induction and maintenance phases of thermal hyperalgesia. (A) When hydrogen water was supplied from day 0 to 4, paw withdrawal latency was longer in the H_2_ (+) group when compared with the H_2_ (–) group (n = 12 mice for each group). Note that the lower threshold in the H_2_ (+) group lasted beyond the termination of hydrogen treatment. (B) When hydrogen water was supplied from day 4 to 21, there was a significant difference in paw withdrawal latency between the H_2_ (+) and the H_2_ (–) groups (n = 12 mice for each group). These experiments were performed concurrently with those in Figure 2. We used a two-way RM ANOVA with a Bonferroni post-hoc test; *P<0.05 vs. contralateral side of same mice. ^#^ *P*<0.05 vs. ipsilateral side of H_2_ (–) mice.

The plantar test also revealed that hydrogen treatment attenuated the induction of hyperalgesia. When hydrogen was administered from day 0 to 4, these mice exhibited significantly longer paw withdrawal latency on the ipsilateral side compared with mice without hydrogen (Figure 4A; two-way RM ANOVA, ipsilateral H_2_ (+) vs. ipsilateral H_2_ (–), *F* = 6.66, *P* = 0.017).

When mice were administered hydrogen water from day 4 to 21, they also exhibited significantly longer paw withdrawal latency on the ipsilateral side compared with that in mice without hydrogen (Figure 4B; two-way RM ANOVA, ipsilateral H_2_ (+) vs. ipsilateral H_2_ (–), *F* = 4.64, *P* = 0.04). These results indicate that hydrogen treatment is effective against both induction and maintenance phases in thermal hyperalgesia.

### Hydrogen Suppressed the Increase of Oxidative Stress in the Spinal Cord Dorsal Horn and the DRG 4 days after PSNL

**Figure 5:**
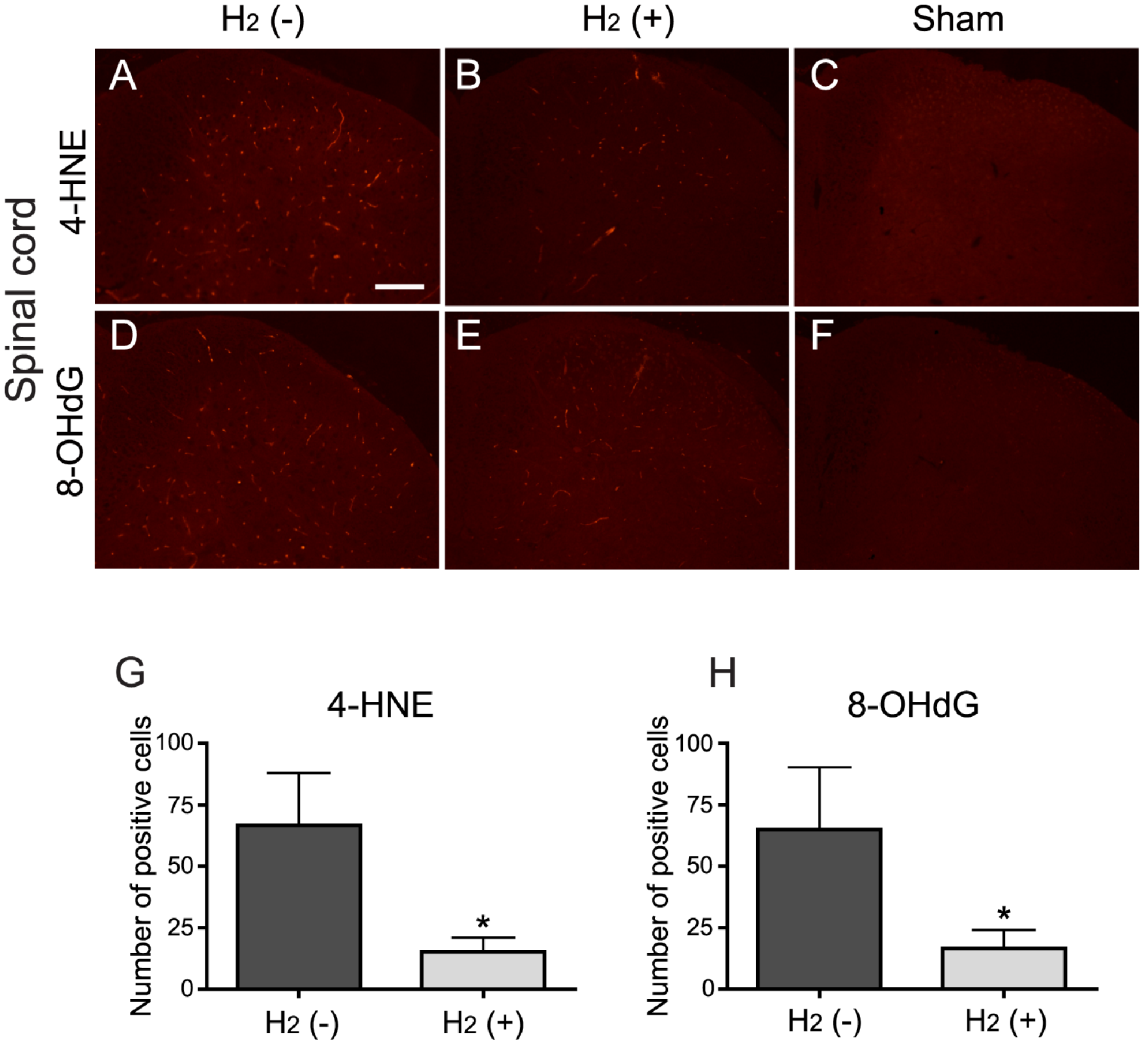
Hydrogen suppressed the development of oxidative stress in the spinal cord dorsal horn. (A–F) Representative images of immunohistochemical staining for the oxidative stress markers 4-HNE (A–C) and 8-OHdG (D-F) in the spinal cord dorsal horn at the level of L5. Staining for 4-HNE was increased in the spinal cord at the end of 4 days period after PSNL (A) compared with sham operation (C). (B) The number of 4-HNE positive cells was decreased in mice with hydrogen when compared with the H_2_ (–) group (A). Similarly, staining for 8-OHdG was also increased in the spinal cord from mice with PSNL (D) compared with sham operation (F). (E) The number of 8-OHdG positive cells was decreased in mice with hydrogen at the end of 4 days period after PSNL when compared with the H_2_ (–) group (D). (G, H) The numbers of positive cells for both markers were significantly decreased when compared with the H_2_ (–) group. *P<0.05 compared with control (*t* test, n = 6 mice for each). Scale bar: 200 µm.

To examine the effect of hydrogen water against oxidative stress, we conducted immunohistochemical analysis using antibodies against oxidative stress markers. We evaluated the numbers of 4-HNE or 8-OHdG positive cells in the spinal cord (at the level of L5) at the end of 4 days period after PSNL or sham operation. The number of 4-HNE positive cells was increased in the spinal cord dorsal horn from mice with PSNL (Figure 5A) compared with the sham-operated controls (Figures 5C). However, the number of positive cells was decreased in mice that received hydrogen water from day 0 (right after PSNL operation) to 4 (Figures 5B) compared with mice without hydrogen (Figures 5A). The quantification confirmed this, indicating a significant difference between them (Figure 5G; *t* test, H_2_ (–) vs. H_2_ (+), t = 2.33, *P* = 0.021). Similarly, the number of 8-OHdG positive cells was increased in the spinal cord dorsal horn from mice with PSNL (Figure 5D) compared with the sham-operated controls (Figures 5F). The number of positive cells was decreased in mice treated with hydrogen (Figures 5E) compared with mice without hydrogen (Figures 5D). The quantification indicated a significant difference between them (Figure 5H; *t* test, H_2_ (–) vs. H_2_ (+), t = 1.83, *P* = 0.049). These results indicate that drinking of hydrogen water suppress the increase of oxidative stress induced by PSNL in the spinal cord dorsal horn.

**Figure 6:**
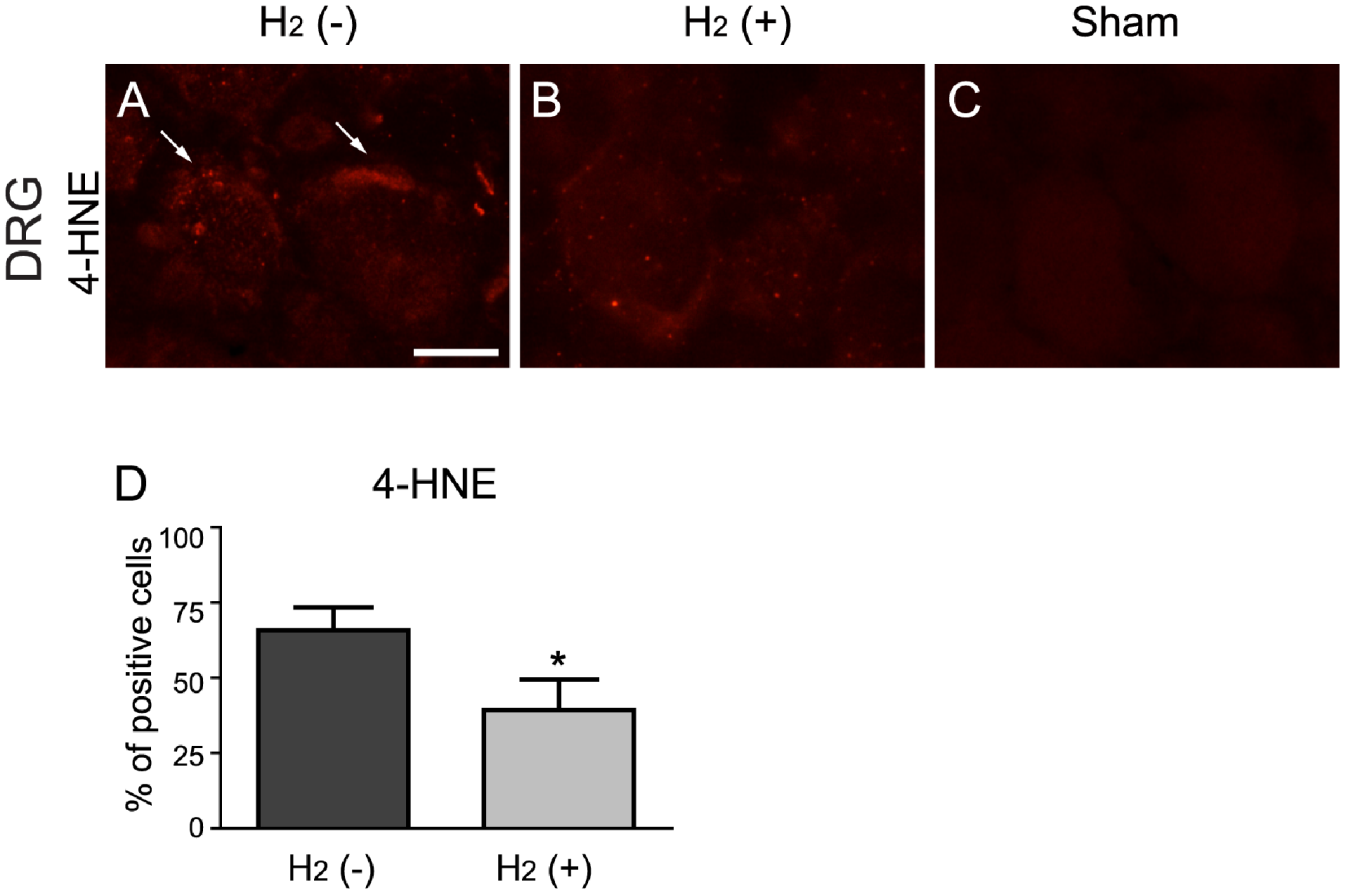
Hydrogen suppressed the development of oxidative stress in the DRG. (A–C) Representative images of immunohistochemical staining for oxidative stress markers 4-HNE in the DRG at the level of L5. Weak staining for 4-HNE (arrows) was observed in the DRG from mice without hydrogen at the end of 4 days period after PSNL (A), whereas staining for 4-HNE was not observed in sham operation mice (C). (B) Staining for 4-HNE was reduced in mice with hydrogen at the end of 4 days period after PSNL. (D) The proportion of 4-HNE positive cells was significantly decreased when compared with the H_2_ (–) group (A). Results are expressed as percent of total neurons. *P<0.05 compared with control (*t* test, n = 6 mice for each). Scale bar: 20 µm.

We also evaluated staining for 4-HNE and 8-OHdG in the DRG (at the level of L5) at the end of 4 days period after PSNL. Weak staining for 4-HNE was detected in mice with PSNL (Figures 6A), but not from the sham-operated controls (Figures 6C). Judging from the morphological appearance, 4-HNE was mainly located in the membrane region of neuronal cells (Figures 6A). Staining for 4-HNE was reduced in mice treated with hydrogen (Figures 6B) compared with mice without hydrogen (Figures 6A). The quantification indicated a significant difference between them (Figure 6D; 4-HNE, *t* test, H_2_ (–) vs. H_2_ (+), t = 2.08, *P* = 0.032). These data indicate that drinking of hydrogen water suppress the increase of 4-HNE induced by PSNL in the DRG. On the contrary, the 8-OHdG positive cells were hardly detected in the DRG from mice with PSNL as well as in those from the sham-operated controls.

**Figure 7:**
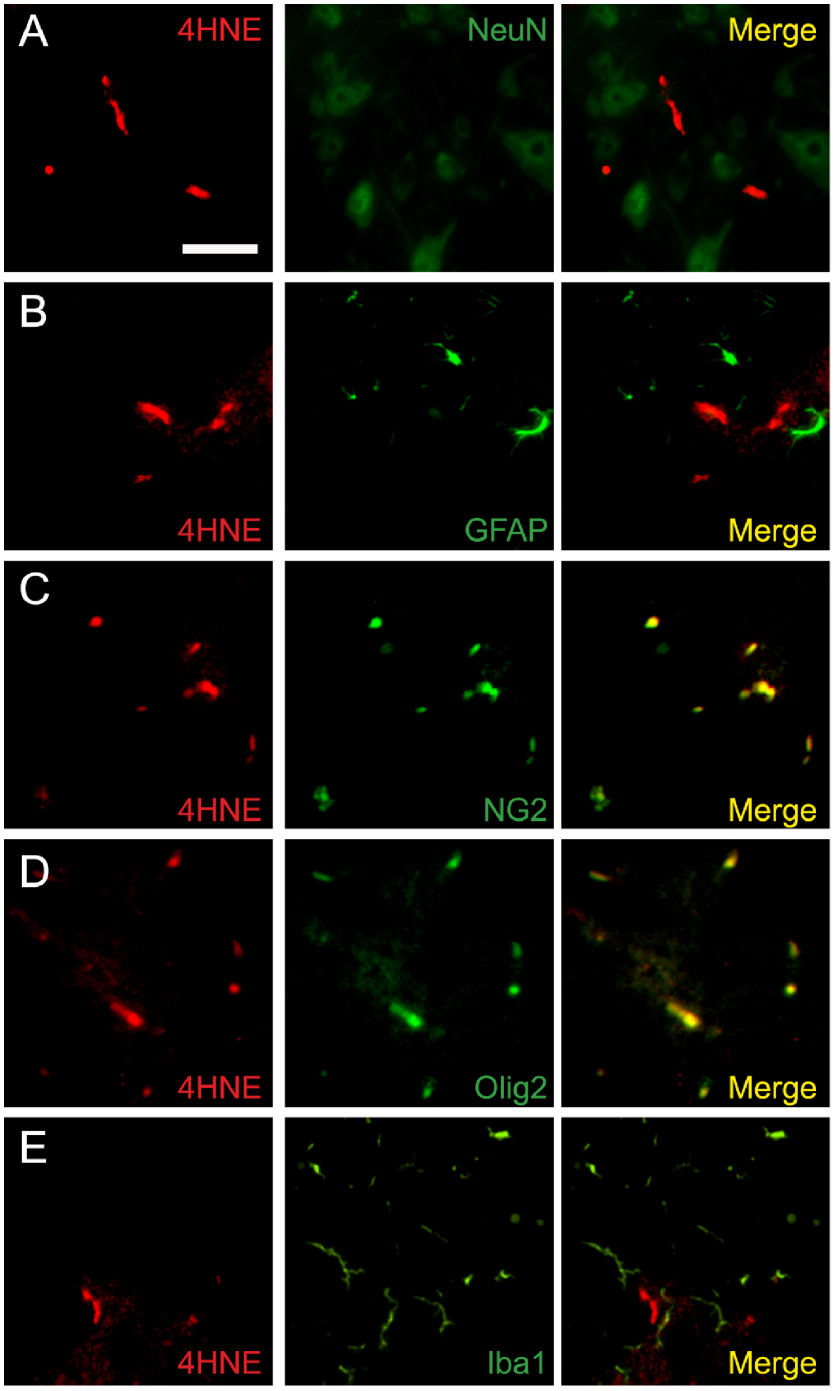
4-HNE were mainly produced on oligodendrocyte in the spinal cord 4 days after PSNL. (A–E) Double immunostaining of 4-HNE with cellular markers for neurons (A), astrocyte (B), oligodendrocyte (C, D), and microglia (E). 4-HNE signals were mainly colocalized with the oligodendrocyte marker NG2 and olig2, but not with NeuN, GFAP, and Iba1 in the spinal cord at the end of 4 days period after PSNL. Scale bar: 20 µm.

We further analyzed the properties of 4-HNE expression in the spinal cord dorsal horn from mice with PSNL at the end of 4 days period after ligation. 4-HNE was hardly detected in neuronal cells, as indicated by double staining for 4-HNE and the neuronal marker NeuN (Figure 7A). Similarly, double staining for 4-HNE and the astrocyte marker GFAP indicated that 4-HNE was not expressed in astrocytes (Figure 7B). On the other hand, double staining for 4-HNE and the oligodendrocyte marker NG2 indicated the expression of 4-HNE in oligodendrocyte (Figure 7C). Furthermore, double staining for 4-HNE and another oligodendrocyte marker Olig2 confirmed the expression of 4-HNE in oligodendrocyte (Figure 7D). Double staining for 4-HNE and the specific microglia marker Iba1 showed that 4-HNE was not expressed in microglia (Figure 7E). These data indicate that 4-HNE is expressed mainly in oligodendrocyte at the end of 4 days period after PSNL.

## Discussion

In this report, we show that hydrogen has a potent analgesic effect against neuropathic pain. Our result is consistent with other previous studies that reported the analgesic effects of ROS scavengers against neuropathic pain. Systemic injection of PBN, which is a non-specific ROS scavenger that scavenges all types of ROS indiscriminately, relieved mechanical allodynia induced by spinal nerve ligation in the rat [5]. Tal et al. reported that systemic injection of TEMPOL reduced hyperalgesia induced by chronic constriction injury in rats [7]. Kim et al. reported that systemic injection of vitamin E attenuated mechanical allodynia and thermal hyperalgesia caused by spinal nerve ligation in rodents [8]. PBN and vitamin E were also effective in producing analgesia by intrathecal injection, indicating that the spinal mechanism is, at least in part, important in neuropathic pain [5], [8]. On the other hand, peripheral ROS also seem to be involved in the generation of pain [1].

We observed that hydrogen reduced oxidative stress caused by PSNL in the spinal cord and the DRG, although little is known about how oxidative stress contribute to neuropathic pain. In this study, we used 4-HNE and 8-OHdG as biomarkers for oxidative stress. 4-HNE is an aldehyde toxic end product of lipid peroxidation and used as a biomarker for membrane lipid peroxidation. 8-OHdG is an oxidized nucleoside accumulated in both mitochondrial and nuclear DNA and used as a biomarker for oxidative DNA damage. 4-HNE and 8-OHdG as well-known biomarkers for oxidative insults caused by hydroxyl radical. Because hydrogen selectively reduces hydroxyl radical [9], 4-HNE and 8-OHdG are suitable for analysing anti-oxidative effect of hydrogen [9]–[11], [20]. In this relation, it was reported that 4-HNE was significantly reduced by drinking hydrogen water in mouse model of Parkinson’s disease, whereas intensity of dihydroethidium, which was oxidized by superperoxide, was not significantly reduced by hydrogen water [11].

Our results indicated that, in the spinal cord, 4-HNE was located mainly in oligodendrocyte at the end of 4 days period after PSNL. Oligodendrocyte is responsible for myelinisation in the CNS and myelin ensheathes neuronal axons allowing fast nerve conduction. Although less is known about the possible role of oligodendrocyte in neuropathic pain, recent report suggests the involvement of spinal oligodendrocyte in neuropathic pain. For instance, spinal transportation of oligodendrocyte progenitor cells reduced spinal cord injury-induced chronic neuropathic pain by enhancing remyelination [21]. Further studies will be needed to to understand the role of 4-HNE in oligodendrocyte for neuropathic pain.

We observed the weak staining for 4-HNE in the DRG at the end of 4 days period after PSNL, which was located in neuronal cells. Staining for 8-OHdG was hardly detected in the DRG in contrary to the spinal cord. Currently, we cannot know the reason for the different expression pattern of markers for oxidative stress between in the DRG and in the spinal cord. Further study is required to understand the role of oxidative stress in the spinal cord and the DRG to neuropathic pain.

We must emphasize that hydrogen was given orally in the current study. Generally, administration of agents by oral pathways is less effective than administration by intra-peritoneal or intrathecal methods. Thus, the efficacy of oral administration is very important from a clinical point of view because continuous intra-peritoneal or intrathecal injections are intolerable. Molecular hydrogen is electrically neutral and has a low molecular weight. Thus, hydrogen readily passes through the blood brain barrier and reaches target cells, unlike other antioxidants that have higher molecular weights and/or polarized structures, making it difficult to reach the target. These specific propensities of hydrogen may contribute to its high efficacy in spite of oral administration. Although we do not know the precise mechanism with which oral hydrogen reduce the increase of oxidative stress after PSNL, oral hydrogen may reach the target site very rapidly. For instance, it was reported that hydrogen molecule can be delivered to the blood in minutes in drinking hydrogen water [10]. It was also suggested that hydrogen might reach the target site by gaseous diffusion, which make it highly effective for reducing hydroxyl radicals [9].

Recently, it was reported that intrathecal administration of hydrogen-rich saline relieve neuropathic pain induced by CCI in rats [22]. This accord with our study and further support the possibility of therapeutic use of hydrogen in pain medicine. Based on the result of this report, anti-oxidant effect of hydrogen in the spinal cord is, at least in part, important in alleviating neuropathic pain. It would be helpful for extending hydrogen application if the date from intraperitoneal injection, intravenous injection, and intrathecal injection of hydrogen was collected as a supporting evidence.

Recently, accumulating evidence suggests that hydrogen is a highly effective antioxidant that has therapeutic potencies for some diseases without remarkable side effects. For instance, hydrogen can reportedly ameliorate reperfusion injury [23], atherosclerosis [24], carcinogenesis [25], neurodegenerative disorders [26], hearing disorders [27], and neuroapoptosis induced by neonatal anesthesia [19] without serious harmful side effect. In addition, hydrogen is already used in clinic for the prevention of decompression sickness in divers [9].

It is important to identify which type of ROS is critically involved in neuropathic pain; however, this is difficult because most antioxidants can react with various ROS indiscriminately. Our results indicate that hydroxyl radical has, at least in part, an important role in both induction and maintenance of neuropathic pain because hydrogen acts only with hydroxyl radical. However, our results could not exclude the possibility that other ROS contribute to neuropathic pain. It was reported that the inhibitors of nitric oxide (NO) synthase reduced pain behaviors, indicating that NO is involved in the mechanism of neuropathic pain [28]. In addition, superoxide is also reportedly involved in the mechanism of neuropathic pain since hyperalgesia was reduced by systemic injection of TEMPOL, which removes one type of free radical, the superoxide [7].

Interestingly, in mice that received hydrogen water from day 0 to 4, the analgesic effect continued even after the cessation of drinking hydrogen water. Therefore, it seems likely that the hydroxyl radical in the induction phase may be important to develop neuropathic pain following PSNL. Furthermore, in mice that received hydrogen water from day 4 to 21, thermal hyperalgesia was gradually improved until day 21, although it reached a maximum at day 4, indicating that the analgesic effect of hydrogen water was also effective against established hyperalgesia. Thus, it may be that hydroxyl radical contributes to maintenance as well as induction of thermal hyperalgesia in the PSNL model. In contrast to hyperalgesia, hydrogen did not improve mechanical allodynia when given during day 4 to 21, indicating that the analgesic effect of hydrogen water was not effective for established mechanical allodynia. Although we do not know the reason for the difference between allodynia and hyperalgesia in the maintenance phase, it is intriguing to speculate that the pathomechanism of mechanical allodynia and thermal hyperalgesia may be different with regard to the vulnerability to oxidative stress in the maintenance phase.

In conclusion, oral administration of water containing hydrogen can be an effective and easy strategy for treating neuropathic pain. Another study indicated that removal of excessive ROS could also produce an analgesic effect against acute inflammatory pain [29]. Further studies will be needed to understand the effect of hydrogen on other types of pain.
